# Brain Plasticity Can Predict the Cochlear Implant Outcome in Adult-Onset Deafness

**DOI:** 10.3389/fnhum.2019.00038

**Published:** 2019-02-19

**Authors:** Ji-Hye Han, Hyo-Jeong Lee, Hyejin Kang, Seung-Ha Oh, Dong Soo Lee

**Affiliations:** ^1^Laboratory of Brain & Cognitive Sciences for Convergence Medicine, Hallym University College of Medicine, Chuncheon, South Korea; ^2^Department of Otorhinolaryngology-Head and Neck Surgery, Hallym University College of Medicine, Chuncheon, South Korea; ^3^Department of Nuclear Medicine, Seoul National University College of Medicine, Seoul, South Korea; ^4^BK21 Plus Global Translational Research on Molecular Medicine and Biopharmaceutical Sciences, Seoul National University, Seoul, South Korea; ^5^Department of Otorhinolaryngology-Head and Neck Surgery, Seoul National University College of Medicine, Seoul, South Korea; ^6^Sensory Organ Research Institute, Seoul National University College of Medicine, Seoul, South Korea; ^7^Department of Molecular Medicine and Biopharmaceutical Sciences, Graduate School of Convergence Science and Technology, Seoul National University, Seoul, South Korea

**Keywords:** cross-modal plasticity, ^18^F-FDG-PET, deafness, cochlear implants, speech perception

## Abstract

Sensory plasticity, which is associated with deafness, has not been as thoroughly investigated in the adult brain as it has in the developing brain. In this study, we examined the brain reorganization induced by auditory deprivation in people with adult-onset deafness and its clinical relevance by measuring glucose metabolism before cochlear implant (CI) surgery. F-18 fluorodeoxyglucose positron emission tomography (^18^F-FDG-PET) scans were performed in 37 postlingually deafened patients during the preoperative workup period, and in 39 normal-hearing (NH) controls. Behavioral CI outcomes were measured at 1 year after implantation using a phoneme identification test with auditory cueing only. In the deaf individuals, areas involved in the auditory pathway such as the inferior colliculus and bilateral superior temporal gyri were hypometabolic compared to the NH controls. The hypometabolism observed in the deaf auditory cortices gradually returned to levels similar to the controls as the duration of deafness increased. However, contrary to our previous findings in congenitally deaf children, this metabolic recovery failed to have a significant prognostic value for the recovery of the speech perception ability in adult CI patients. In a broad occipital area centered on the primary visual cortices, glucose metabolism was higher in the deaf patients than the controls, suggesting that the area had become visually hyperactive for sensory compensation immediately after the onset of deafness. In addition, a negative correlation between the metabolic activity and behavioral speech perception outcomes was observed in the visual association areas. In the medial frontal cortices, cortical metabolism in most patients decreased, but patients who had preserved metabolic activities showed better speech performance. These results suggest that the auditory cortex in people with adult-onset deafness is relatively resistant to cross-modal plasticity, and instead, individual traits in late-stage visual processing and cognitive control seem to be more reliable prognostic markers for adult-onset deafness.

## Introduction

Cochlear implants (CIs) have provided restoration of hearing to people with severe to profound hearing loss. Hearing restoration with CI induces changes in the way sound inputs are processed in the brain. Brain reorganization is induced by deafness and subsequent hearing restoration *via* the CI and is not limited to just the auditory cortex but also includes other areas, including the visual cortex (Stropahl et al., 2017). This cross-modal plasticity seems particularly pronounced in CI users whose language comprehension is dependent on visual cues such as speechreading (Strelnikov et al., 2013). In CI users, reduced activation in the visual cortex and increased activation in the auditory cortex in response to visual stimuli has been found (Sandmann et al., 2012; Chen et al., 2016). Studies using F-18 fluorodeoxyglucose positron emission tomography (^18^F-FDG-PET) have also found that a time-dependent increase in the metabolism in the auditory areas was correlated with the duration of deafness both in prelingually and postlingually deafened persons (Lee et al., 2001, 2003, 2007a). Moreover, in prelingually deafened individuals who have a greater cross-modal activity between the visual and auditory cortices, brain activation in the visual cortex in response to visual stimuli was associated with speech perception outcome after CI surgery (Finney et al., 2001; Lee et al., 2001, 2007b; Levänen and Hamdorf, 2001). Such neurophysiologic evidence and related clinical findings reinforce the importance of early CI surgery and rehabilitation for congenitally deaf children in order to facilitate normal language development (Yoshinaga-Itano et al., 1998; James and Papsin, 2004; Sharma et al., 2005). Recent studies using high-density electroencephalogram (EEG) and functional near-infrared spectroscopy (fNIRS) have shown the higher activation in the auditory cortex in response to visual stimuli, and that the greater activation in the area was associated with the better behavioral performance in postlingually deafened CI users (Stropahl et al., 2015; Chen et al., 2016; Anderson et al., 2017). These results suggest that the cross-modal reorganization driven by strong audiovisual connectivity is not a maladaptive effect, rather a positive result of the brain plasticity due to the hearing restoration after CI use.

Perhaps the most important current issue in CI research is that speech and language outcomes are tremendously variable across CI recipients. There is limited biomarker for measuring CI outcome, but having such a marker may help to understand the sources of outcome variability. Having a biomarker is even more important for pediatric CI users whose speech perception may not be easily evaluated by behavioral measures. However, currently used objective measures such as the stapedius reflex, electrically evoked compound action potentials, or electrically evoked auditory brainstem responses have shown poor relationships with speech perception (Abbas and Brown, 1991; Hirschfelder et al., 2012; Lundin et al., 2015). Among demographical factors, the duration of deafness has been correlated with the speech perception abilities of CI users. However, results on the correlations are inconsistent, thereby showing limited predictive value (Oh et al., 2003; Roditi et al., 2009). Unlike these peripheral measures and demographic factors, cross-modal brain plasticity measured by the degree and location of brain activity has revealed significant relationships with the speech perception abilities in both pre- and postlingually deafened CI users (Lee et al., 2001, 2003, 2007a; Sandmann et al., 2012; Strelnikov et al., 2013; Han et al., 2016). For example, brain activation in the visual cortex elicited by a speech-processing task in postlingually deafened adults was positively related to CI speech outcomes (Strelnikov et al., 2013). In this study, the greater degree of cross-modal plasticity indicated better speech recovery in the CI users.

The audiovisual cross-modal plasticity compared to the processing of auditory or visual information has been extensively studied in prelingually deafened individuals and prelingually deafened CI users (Finney et al., 2001; Lee et al., 2001; Karns et al., 2012; Hauthal et al., 2013; Bottari et al., 2014; Shiell et al., 2014). Nevertheless, it is uncertain whether the functional takeover pattern in postlingually deafened individuals is similar to that observed in prelingually deafened people. Given that postlingually deafened people acquire deafness after the full development of the auditory system and previously had a normal language function, the pattern and degree of brain plasticity in these individuals might be different from those in prelingually deafened people. It is also known that postlingually deafened individuals are much more dependent on lipreading to compensate for the deprived hearing sense compared to prelingually deafened people whose primary communication tool is sign language (Lee et al., 2007b; Suh et al., 2009).

These behavioral distinctions between the two deaf groups have been reflected in neurophysiological findings such that postlingually deafened individuals have enhanced neural networks and faster processing to speechreading compared to prelingually deafened people (Lee et al., 2007a; Suh et al., 2009). Moreover, brain reorganization in postlingually deafened persons involves variable neural networks outside of the auditory cortex, including language networks or networks underlying visuospatial attention, while the multisensory integration binding auditory-language areas are weakened in prelingually deafened individuals (Giraud et al., 2001; Bavelier et al., 2006; Suh et al., 2009).

**Figure 1:**
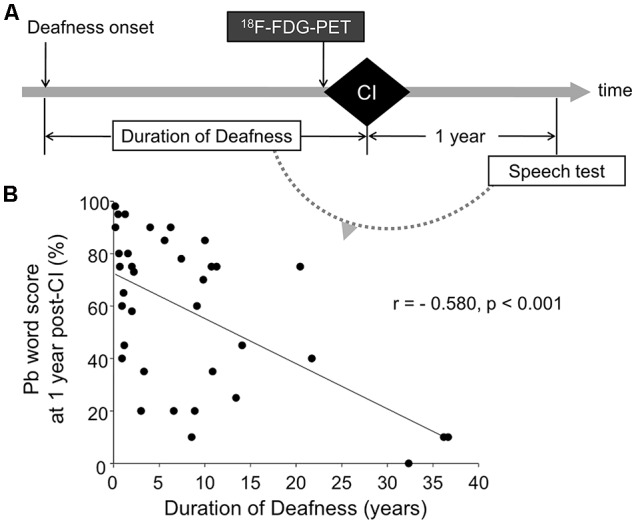
The experimental paradigm **(A)** and correlation between deafness duration and cochlear implant (CI) speech outcome **(B)**. F-18 fluorodeoxyglucose positron emission tomography (^18^F-FDG-PET) was performed prior to CI surgery. Imaging data were correlated with the duration of deafness, and speech scores measured at 1 year post-CI. CI speech scores were significantly correlated with deafness duration (*r* = −0.580, *p* < 0.001).

At present, a few studies have examined the visual takeover of the auditory cortex in postlingually deafened people before acquiring a CI, and the findings are inconsistent. For instance, the visual takeover in the auditory cortex in postlingually deafened individuals was negatively correlated with CI speech outcomes (Doucet et al., 2006; Sandmann et al., 2012; Kim et al., 2016) whereas other studies have reported that greater cross-modal reorganization was related to better recovery after CI surgery (Rouger et al., 2012; Strelnikov et al., 2013). More studies are needed to determine whether cross-modal plasticity is a positive indicator of the outcomes of patients with CIs. Thus, in the present study, we measured the resting state metabolism using ^18^F-FDG-PET scans from 37 postlingually deafened adults and 39 age- and gender-matched normal-hearing (NH) controls before CI surgery. To determine whether the neurophysiological changes are related to speech outcomes for the CI patients, we examined relationships between speech scores measured 1 year after implantation and their resting brain metabolism (Figure 1A). We hypothesized that cross-modal plasticity in the visual and auditory cortices would be revealed in CI users, but the brain area involving higher metabolism in CI users would be different from those in NH controls due to the plasticity. In addition to the altered metabolic activity, relationships between the activities and speech perception abilities in the postlingually deafened CI users were also hypothesized.

## Materials and Methods

### Subjects

Thirty-seven postlingually deafened patients [24 women; mean age (±SD) = 44.5 (±11.4) years; age range = 22–60] participated. All of the subjects had no history of neurologic and/or psychiatric disorders and were right-handed. Table 1 contains the demographic data for the CI and NH groups. The CI subjects had bilateral severe-to-profound sensorineural hearing loss and were classified as CI candidates. The etiology of the hearing loss included idiopathic progressive sensorineural hearing loss (*n* = 20), sudden sensorineural hearing loss (*n* = 9), chronic otitis media (*n* = 6), and other causes (*n* = 2). The duration of deafness was counted from the onset of profound hearing loss, which was defined as the time when oral communication became impossible even with a well-fitted hearing aid. In many cases, the duration of hearing loss is not reliable since it is more attributed to subjective memory rather than medical needs. Subsequently, we took the duration of deafness rather than the duration of hearing loss as a factor representing the duration of auditory deprivation. Among six patients who self-reported the onset of hearing loss during their mid-teenage years, four lost hearing completely in their late teens and the other two after the age of 20. For the rest, deafness started either progressively or after the age of 20. Patients with deafness onset during childhood were excluded to maintain the homogeneity of the study population. This inclusion criterion ensures that the deafness was initiated after the full development of the brain, including phonetic and semantic areas for normal auditory language function. All of the deaf subjects were fluent in Korean as well as speechreading, but did not use sign language before implantation. The duration of deafness varied from 2 months to 37 years [mean (±SD) = 8.4 years (±9.9 years)]. ^18^F-FDG-PET scanning was performed during presurgical examination prior to cochlear implantation (25 Cochlear Co., 9 Advanced Bionics, and 3 Med El devices). One year after CI switch-on, behavioral speech perception was measured using a word perception test. In this test, a list of 25 phonetically balanced (pb) words was presented verbally with no visual cue, and the percent correct was calculated. Speech scores were obtained from all of the CI participants except for one (*n* = 36). Figure 1A shows the overall design of the current study.

**Table 1:** Demographic data for CI and normal-hearing (NH) groups.

	Normal hearing	CI users
Age of test	47.5 (±13.2)	44.5 (±11.4)
Duration of deafness		8.3 (±9.7)
Duration of hearing loss		16.5 (±12.4)
Pb word scores (%)		58 (±29.2)

*Values are mean ± SD. CI, cochlear implant; Pb, Phonetically-balanced*.

^18^F-FDG-PET scans for 39 age- and gender-matched NH controls [24 women; mean age (±SD) = 47.5 (±13.2) years; age range = 20–67] were obtained from the internal database of the PET center of the Department of Nuclear Medicine at Seoul National University Hospital. There was no difference in age between the deaf and NH groups (*t*_(76)_ = −1.01, *p* = 0.31). All of the NH participants were right-handed. The audiometric hearing thresholds of the NH subjects were less than 20 dB hearing level (HL) for octave test frequencies from 250 to 8,000 Hz. None of the subjects reported a history of neurologic, psychiatric, or otologic disorders, and informed consent was obtained from all of them. The study protocol was approved by the Institutional Review Board of the Seoul National University Hospital. All subjects gave written informed consent in accordance with the Declaration of Helsinki.

### FDG-PET Scanning

^18^F-FDG-PET scans were performed before CI surgery using an ECAT EXACT47 (Siemens-CTI, Knoxville TN, USA) PET scanner (BGO crystal detector, sensitivity 214 kcps/microCi/min) in two-dimensional mode with a 16.2 cm axial field of view. Subsequently, 370 Mbq or less of ^18^F-FDG was injected intravenously 30 min before scanning. Between the injection and the scanning, the subjects remained in a waiting room with ambient light and noise and without any specific instructions. A transmission scan was performed using a Ga-68 rod source to yield attenuation maps immediately before the emission scan, during which 47 slices of emission images were acquired over a 20-min period. These were then reconstructed in a 128 × 128 × 47 matrix with a pixel size of 2.1 × 2.1 × 3.4 mm using a filtered back projection method with a Shepp filter using a cutoff value of 0.35 cycle/pixel. All of the reconstructed images were corrected for attenuation, and the trans-axial images were realigned to produce sagittal and coronal images.

### Imaging Analysis

The SPM8 package (Institute of Neurology, University College of London, UK) implemented in Matlab R2008a (Mathworks Inc., Natick, MA, USA) was used for image preprocessing (realignment, spatial normalization, and spatial smoothing with a 16 mm full-width at half maximum Gaussian kernel) and statistical analysis.

In order to examine the metabolic differences between the deaf patients and the NH controls, a full-factorial design with group as a factor and age as a covariate was created. Statistical significance was set at *p* = 0.05, and family-wise error (FWE) rate correction was applied for multiple comparisons (*T* = 4.43). A bivariate linear regression analysis was performed on the duration of deafness and the word recognition scores to determine whether the former as a demographic factor could predict the CI outcome. The Pearson product-moment correlation coefficient was applied to measure changes in brain metabolism as a function of the duration of deafness in the deaf patients. For those whose speech scores were obtained at 1 year post-CI surgery, an additional correlation analysis between the metabolic activity and the speech scores was performed to determine which brain regions are related to the post-CI speech outcome. The statistical significance of the correlation analyses was set at *p* = 0.001, uncorrected (*T* = 3.35 with the deafness duration and *T* = 3.36 with the speech scores). For all of the analyses outlined, the effect of age was factored out by considering it as a nuisance variable.

## Results

### Clinical Data: The CI Speech Outcome and the Duration of Deafness

The speech scores after CI surgery varied from 0 to 98%. A linear regression analysis showed a significantly negative correlation of speech outcome with the duration of deafness (Figure 1, *r* = −0.580, *p* < 0.001), indicating that individuals with lower speech perception scores had longer duration of deafness than individuals with higher speech perception scores. However, the speech outcome results revealed a great deal of variability, particularly for subjects who had a relatively short duration of deafness. In the subset of the deaf patients whose deafness duration was equal to or less than 10 years (*n* = 27), their speech scores were not significantly correlated with the duration of deafness (*r* = −0.294, *p* = 0.146).

### Group Comparison

**Figure 2:**
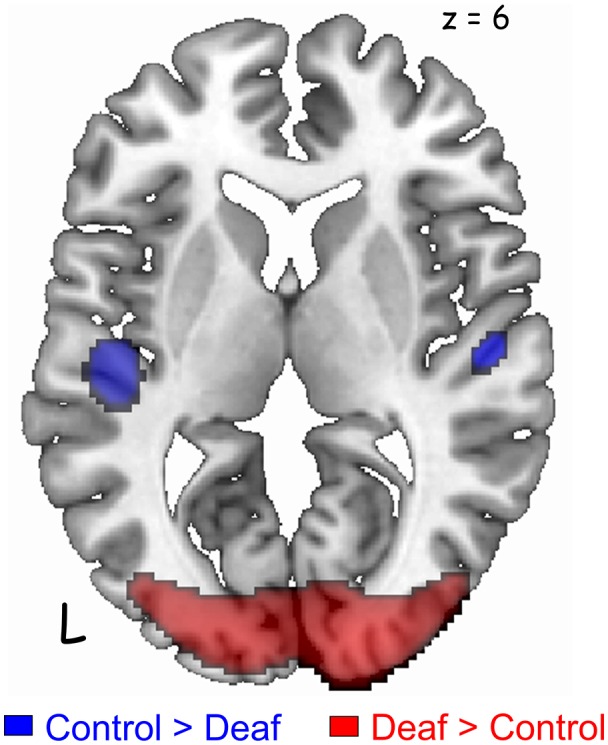
Significant differences in the auditory and visual cortices between the groups [*p* < 0.05, family-wise error rate (FWE)-corrected for multiple comparisons]. Deaf patients showed decreased metabolism in the bilateral auditory cortices (blue) and increased metabolism in the visual cortex (red). A cluster in the right auditory cortex survived after small volume correction (SVC) with a corrected *p*-value of 0.001.

The deaf group showed decreased metabolism in regions of the auditory pathway, including the inferior colliculus and the bilateral superior temporal gyrus (Table 2, Figure 2). However, the metabolism in the right temporal gyrus of the deaf patient group was not significantly different with the NH group (40 voxels with uncorrected *p* = 0.0005), but survived following a small volume correction (SVC; 5 mm sphere at peak voxel, FWE-corrected *p* = 0.001). For the deaf group, the cerebral glucose metabolism was significantly higher in the bilateral calcarine gyri and the right posterior middle temporal gyrus (BA 39), while the metabolism was lower in areas of the bilateral dorsal cingulate gyri (Table 2).

**Table 2:** Group comparisons (family-wise error, FWE-corrected *p* = 0.05, *T* = 4.43, extent threshold *k* = 10).

	R/L	Brain region (BA)	MNI coordinates	Cluster size	Voxel T
Normal > Deaf	L	Superior temporal gyrus (41, 42)	−46 −24 16	763	4.97
	*R*	*Superior temporal gyrus (41)*	*54 −14 6*	*40 (up.0005)*	*3.59 (SVC)*
		Inferior colliculus	0 −36 −14	12	4.26
	R	Cingulate gyrus (24)	22 6 36	1425	6.55
	L	Cingulate gyrus (24)	−12 −4 36	1347	5.31
Deaf > Normal	R	Calcarine gyrus (17, 18)	18 −94 −4	6974	7.47
	L	Calcarine gyrus (17, 18)	−14 −94 −8	within above cluster	7.26
	R	Middle temporal gyrus, posterior (39)	62 −60 16	58	4.69

*SVC; small volume correction*.

### Correlation Analyses With the Duration of Deafness

**Figure 3:**
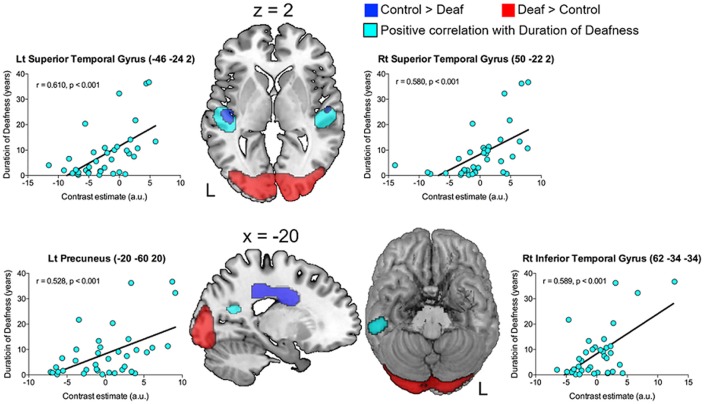
The brain regions showing significant correlations with clinical factors (*p* = 0.001, uncorrected). The images are overlaid with the results of the group comparison in Figure 2 (blue: controls > deaf, red: deaf > controls). A gradual increase in metabolism as a function of deafness duration (sky blue) was found in the bilateral superior temporal gyri, the left precuneus, and the right inferior temporal gyrus.

**Figure 4:**
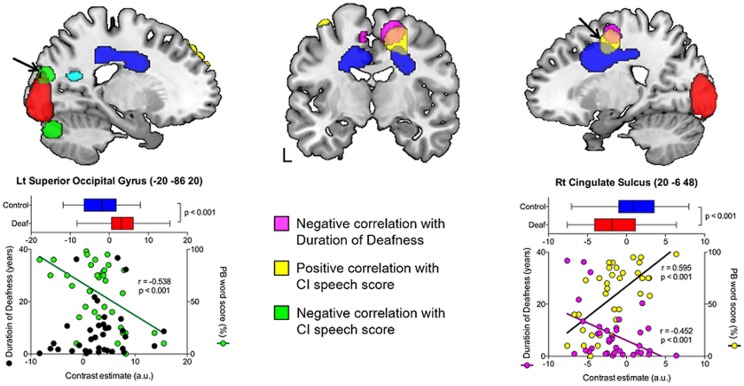
The occipital and cingulate cortex activity in the correlation analysis with duration of deafness and CI speech score. A negative correlation with CI speech outcome (green) was found in the left superior occipital gyrus, for which the metabolism did not show a significant temporal change. A positive correlation with CI speech (yellow) was found in the lateral prefrontal regions and in the right cingulate sulcus. The latter overlapped with an area of hypometabolism in deafened individuals compared to the controls (blue) and time-dependent decrease in metabolism as a function of deafness duration (pink).

Correlation analyses were carried out to examine whether glucose metabolism in the areas of interest has a relationship with any of the demographical factors in adult-onset CI users. As shown in Figure 3 and Table 3, the results revealed that the metabolism in the left and right superior temporal gyri was significantly increased as the duration of deafness increases (Lt: *r* = 0.610, *p* < 0.001; Rt: *r* = 0.580, *p* < 0.001). These clusters overlapped with areas where a group difference between CI and NH was evident (CI < NH). Positive correlations between the metabolism and deafness duration were also found for the right inferior temporal gyrus (*r* = 0.589, *p* < 0.001) and the left precuneus (*r* = 0.528, *p* < 0.001). Meanwhile, the glucose metabolic activity was negatively correlated with the duration of deafness in the bilateral medial frontal cortices (*r* = −0.452, *p* < 0.001), which occupy the cingulate sulcus. These areas are in the vicinity of the regions where the metabolism was lower in the deaf patient group than in the NH control group (Figure 4, middle and right).

**Table 3:** Regions showing a significant correlation between glucose metabolism and duration of deafness (uncorrected *p* = 0.001, *T* = 3.35).

R/L	Brain region (BA)	MNI coordinates	Cluster size	Voxel T
Increased metabolism with duration of deafness				
L	Superior temporal gyrus (22)	−46 −24 2	642	4.52
R	Superior temporal gyrus (22)	50 −22 2	518	4.18
R	Inferior temporal gyrus (37)	62 −34 −34	112	4.28
L	Precuneus	−20 −60 20	69	3.65
Decreased metabolism with duration of deafness				
R	Cingulate sulcus (6)	14 −8 56	505	4.43
L	Cingulate sulcus (6)	−8 −12 52	65	3.59

### Correlation Analyses With CI Speech Scores

**Figure 5:**
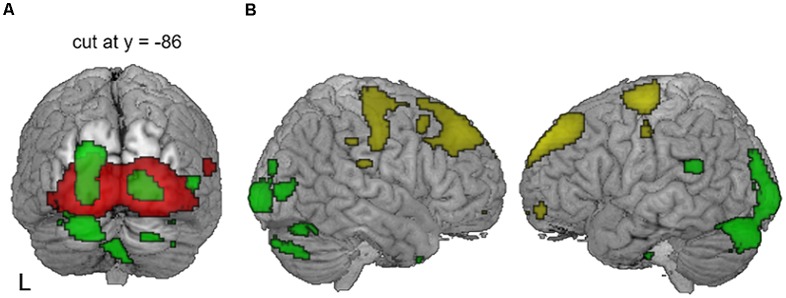
Patterns of CI outcome-related metabolism with lenient thresholds. **(A)** With compensatory visual hyperactivity centered in the bilateral calcarine sulci (red), speech-negative correlation (green) centered on the left superior occipital gyrus extended to the right secondary visual area when a lenient statistical threshold was used (uncorrected *p* = 0.005) but avoided the primary areas along the midline. **(B)** With an even more lenient threshold of *p* = 0.01 (uncorrected), dorsoventral dissociation of the metabolic pattern was still apparent. Regions where metabolism was related to good (yellow) vs. poor (green) CI outcomes were located separately in the dorsal frontal vs. the ventral occipitotemporal areas.

As shown in Table 4, positive correlations were found between the metabolism in the dorsolateral and dorsomedial frontal areas and the word scores measured a year after CI surgery. Pearson’s correlation coefficient analysis confirmed that the metabolism in the right cingulate sulcus increased as CI speech scores increased (*r* = 0.595, *p* < 0.001). A cluster with a peak voxel in the right cingulate sulcus overlapped with regions of hypometabolism in the deaf patients compared to the NH control group. In contrast, the CI speech scores increased with the metabolism decrease in the left superior occipital gyrus (*r* = −0.538, *p* < 0.001) where clusters occupy a part of the region showing hypermetabolism in the deaf patients. In addition, when using a lenient threshold (uncorrected *p* = 0.005), the area showing a negative correlation extended to the secondary visual association areas in both hemispheres but not the primary visual area near the midline (Figure 5A). If we applied an even more lenient threshold (uncorrected *p* = 0.01), the brain areas were divided into two parts: the dorsal frontal cortex and the ventral occipitotemporal regions, showing positive and negative correlations with the CI speech scores, respectively.

**Table 4:** Correlation analysis in deaf group with CI speech score at 1 year (Pb word test word score under auditory only condition; uncorrected *p* = 0.001, *T* = 3.35).

R/L	Brain region (BA)	MNI coordinates	Cluster size	Voxel T
Positive correlation with speech score				
L	Superior frontal gyrus (8)	−18 56 40	303	4.78
R	Cingulate sulcus (24)	20 −6 48	490	4.65
L	Precentral gyrus (4)	−44 −14 66	144	4.27
R	Middle frontal gyrus (8)	38 40 46	54	3.55
Negative correlation with speech score				
L	Cerebellum	−28 −76 −26	394	3.97
L	Superior occipital gyrus (19)	−20 −86 20	189	3.89

## Discussion

**Figure 6:**
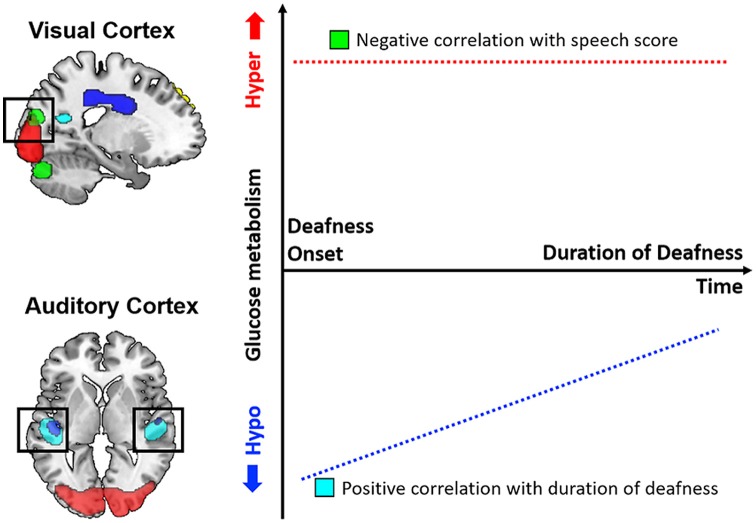
Schematic diagram of glucose metabolic activity in the visual and auditory cortices and its correlation with speech perception results. In postlingually deafened subjects, metabolic activity was higher in the visual cortex and lower in the auditory cortex compared to the normal-hearing (NH) controls. In addition, the metabolic activities in the auditory areas were significantly correlated with the duration of deafness, but no correlation with speech perception was discovered. In contrast, a negative correlation between the metabolic activities and speech scores was revealed for the visual area.

In the present study, glucose metabolism was measured using ^18^F-FDG-PET scans before CI surgery for 37 postlingually deafened adults in order to determine any correlations with the duration of deafness as well as post-CI speech perception ability. In comparison with an NH control group, the metabolism was lower in the area including the auditory pathway but higher in the visual areas for the deaf patients. In a correlation analysis, the results showed that the metabolism in the auditory regions was positively correlated with the duration of deafness, while the metabolic activity in the occipital area was negatively correlated with the CI speech outcome. In addition, a correlation analysis between the metabolism and speech performance indicated that patients with higher metabolic activities in the cingulate sulcus had better CI speech outcomes. A schematic diagram of the results is depicted in Figure 6. In sum, our results indicate that the cross-modal plasticity is initiated with the deafness onset. However, the plasticity patterns were distinct among brain areas. In the visual cortex, metabolism was not changed with the increase in duration of deafness, whereas the auditory cortex metabolism changed as a function of the deafness duration. Given that the speech scores were lower in CI users with higher visual cortex metabolism, the maladaptive audio-visual takeover was revealed in this study.

### Deafness-Induced Plasticity in the Auditory Cortex

In the current study, the glucose metabolism in the auditory cortices of deaf patients decreased compared to the NH group, and the metabolism in deaf patients with longer duration of deafness was lower than that in patients with shorter duration of deafness. The increase in metabolism with time in the hypometabolic auditory areas has previously been reported both in prelingually and postlingually deafened people (Lee et al., 2001, 2003, 2007a) and animals (Ahn et al., 2004; Park et al., 2010). For instance, Lee et al. (2001) reported the first evidence of metabolic changes as a function of deafness duration, and the time-dependent metabolic activity increase in the deprived auditory cortex was attributed to audiovisual take-over due to cortical plasticity. It is well-known that in deafness, the auditory cortices are activated for other sensory processing involving the visual or vibrotactile senses due to cross-modal reorganization (Puce et al., 1998; Finney et al., 2001; Levänen and Hamdorf, 2001; MacSweeney et al., 2004; Fine et al., 2005; Doucet et al., 2006; Obretenova et al., 2010; Buckley and Tobey, 2011; Sandmann et al., 2012; Stropahl et al., 2015, 2017; Chen et al., 2016; Anderson et al., 2017). Indeed, in our study, the glucose metabolic rate in the left and right superior temporal gyri increased with an increase in deafness duration. This finding is consistent with a previous report by Kral et al. (2003) in that the peak voxels of time-dependent plasticity were found in the bilateral secondary auditory areas but not in the relatively hard-wired primary auditory cortex. Thus, we assumed that the non-auditory network was reorganized in order to improve the processing of auditory information in postlingually deafened individuals. Previous findings that the non-speech network was recruited to assist speech processing during an auditory imagery task in a subgroup of postlingually deafened patients may support our assumption (Lazard et al., 2011, 2013).

In our previous study examining prelingually deafened patients, the cross-modal reorganization in the auditory cortices was shown to be related to speech perception ability, which indicates its clinical relevance when predicting CI outcome (Lee et al., 2007a). Once the functional takeover between the auditory and visual cortices occurs, the responsiveness and/or efficiency of auditory processing markedly decreases. Consequently, when the auditory cortex of deaf persons has less hypometabolism, their speech perception skills with CI use do not recover as well as people whose auditory cortex was not taken over by other sensory modalities (Lee et al., 2001, 2007a). Contrary to prelingually deafened individuals, the negative effect of the functional takeover on CI speech outcome was not found in the current study, which might have been because the development of the auditory pathway of postlingually deafened individuals is different from that of prelingually deafened people. In adult-onset deafness, the auditory pathway might be intact because auditory deprivation is initiated after the central auditory system has already fully developed (Petersen et al., 2013). Furthermore, an intact pathway seems to be maintained even if the auditory cortices are engaged in the processing of other senses *via* disinhibition of latent cortico-cortical connections (Bavelier and Neville, 2002; Chabot et al., 2015). Thus, it is possible that the original function of the auditory cortex will be easily recovered by reintroducing acoustic signals through CIs. The effective regeneration system in the auditory cortex in postlingually deafened CI users may interfere the maladaptive of cross-modal plasticity previously shown in prelingual deafness (Lee et al., 2007a). However, relatively few studies have been published for postlingually deafened CI users that examine relationships between the auditory cortex and speech perception. Thus, the debate on the functionality of cross-modal reorganization is still ongoing. In the maladaptive view, higher activation for visual processing in the auditory cortex was negatively related to speech performance (Sandmann et al., 2012). On the other hand, positive relationships between the auditory cortex activation to visual stimuli and behavioral speech scores were found (Stropahl et al., 2015, 2017 for review). More studies are needed to determine the functionality of cross-modal plasticity in the auditory cortex in postlingually deaf CI users.

A study comparing cortical responses of post- and prelingually deafened subjects has shown that the amplitudes of cortical evoked potentials in response to simple visual stimuli recorded from temporal sites were related to the CI outcome in prelingually but not in postlingually deafened subjects (Buckley and Tobey, 2011). Similarly, a recent PET study has also offered evidence that the plastic changes between pre-and postlingually deafened CI recipients are different (Petersen et al., 2013). In the study, postlingually deafened CI users revealed greater activation of speech over babble in the bilateral middle and superior temporal gyri, indicating that the postlingually deafened CI users can selectively differentiate meaningful speech from babble whereas the prelingually deafened CI recipients cannot. The adult-onset deafened patients with mature brains would have a distinct pattern of the reorganization involving the auditory cortex compared with congenitally deafened individuals whose auditory pathways had not yet undergone normal development. Nonetheless, a recent finding stands for the opposite results from our study, such that in congenitally deaf cats the auditory-responsive neurons were largely intact even though the cross-modal plasticity has been taken place due to the deafness (Land et al., 2016). More studies are needed to understand the degree, causes, and patterns of cross-modal reorganization in relation to the duration and onset of deafness.

The glucose metabolic rate of the higher visual associative cortices including the right inferior temporal gyrus and precuneus was positively correlated with the duration of deafness. This result suggests that vision is superior to other sensory modalities in the functional takeover of the auditory sense. It also indicates that the visual associative cortices have a role in processing auditory information and multimodal integration. In fact, the inferior temporal gyrus and the precuneus are known to process auditory speech and auditory integration for CI users. For example, the precuneus has been associated with the processing of visuospatial imagery and audiovisual integration (Hertz and Amedi, 2010), and the inferior temporal gyrus has been related to extracting information from multimodal inputs and auditory speech processing in CI users (Giraud and Truy, 2002; Xu et al., 2009).

### Deafness-Induced Plasticity in the Visual Cortex

Once the hearing sense has been deprived, postlingually deafened individuals spontaneously become dependent on their intact senses, such as vision, and this behavioral change is reflected in brain functional changes measured by neuroimaging techniques. For example, previous PET studies have demonstrated that visual-auditory cross-modal reorganization occurs in deaf individuals, and that the degree of reorganization is associated with auditory language skills (Lazard et al., 2010; Strelnikov et al., 2013). Similarly, for the deaf groups in the current study, the resting state brain activity in the visual areas increased and the brain activity was related to behavioral speech perception. It was assumed that the deaf patients were visually hyperactive in order to receive visual information to compensate for the deprivation of auditory information regardless of whether stimulation was ongoing or not. Furthermore, this finding is related to the behavioral characteristics of postlingually deafened patients who are known to rely considerably on speechreading to enhance auditory speech understanding. This is because the linguistic structure of the speechreading is similar to that of previously used auditory speech, while other visual languages such as sign language is different (Lee et al., 2007b; Strelnikov et al., 2013; Lazard et al., 2014).

Interestingly, a significant difference in glucose metabolism between the deaf and NH groups was found in the bilateral primary visual areas, although the areas relating to CI speech outcome were the visual associative areas and cerebellum rather than the primary visual cortex. Even with a lenient threshold, outcome-related areas were spread out over the bilateral visual associative cortices but not the calcarine sulcus in the midline (see Figure 5A). These findings indicate that although the glucose metabolism in postlingually deafened patients is hyperactive during visual processing, the behavioral benefits associated with CI are determined by how they process what they see. In turn, this means that cognitive involvement in visual processing can limit the CI speech outcome. Previous studies examining the brain activity of visual stimulation in deaf subjects have failed to demonstrate a general enhancement of the visual function (Finney and Dobkins, 2001; Brozinsky and Bavelier, 2004). For instance, no differences have been found in the sensitivity to stimuli with varied visual contrasts nor in the visual field asymmetry between deaf and hearing subjects. In those studies, the authors suggested that deafness does not alter visual processing ability, rather attention changes the visual perception through attention-to-motion processes (Emmorey et al., 1993; Bavelier et al., 2006; Dye et al., 2008). Therefore, we assume that the functional reorganization in postlingually deafened individuals occurs in the focal visual associative cortex for the enhanced visual processing *per se*. Moreover, the reorganization includes the visual associative area involved in cognitive/language processing controlled by the top-down modulation of the higher-order cortex (Bavelier et al., 2006). However, not many studies have been conducted to confirm this theory, thus further ones are warranted to clarify this issue.

### Deafness-Induced Plasticity in the Medial Frontal Cortex

The glucose metabolism in the immediate vicinity of the medial frontal cortex was significantly different between the deaf and NH groups, and correlated with two clinical factors: deafness duration and post-CI speech outcome. In the dorsal cingulate cortex, most CI patients had decreased metabolism, regardless of the onset of deafness, while the reduced metabolism in the cingulate sulcus more varied depending on the period of deafness. In addition, an overlapping area between the dorsal cingulate cortex and the cingulate sulcus was related to post-CI speech performance. These results indicate that metabolism in these areas decreases after deafness onset at different paces, and hypometabolism in this area was significantly related to the limited recovery of auditory performance after CI surgery.

Until recently, the role of the cingulate cortex was not fully understood. Since this area is close to the area governing motor control, it is believed that a major role of the cingulate cortex is the integration of the senses and self-motion (Cardin and Smith, 2011; Fischer et al., 2012). Meanwhile, emerging evidence suggests that the cingulate sulcus is strongly responsive to visual and vestibular inputs, especially at the cingulate sulcus visual (CSv) area (Fischer et al., 2012; Smith et al., 2012). Because the location of the CSv area is at a distance from the visual and vestibular areas but close to the region for motor control, we assume that it delivers sensory information to the motor system. To evaluate this assumption, a recent study was conducted to examine the connectivity pattern of the CSv area using resting-state and diffusion MRI (Smith et al., 2018). In this study, the researchers found a strong connection between it and the motor areas including the supplement motor area (SMA), indicating that the CSv has a critical role in transferring visual information for self-motion into the cingulate motor system (Smith et al., 2018). Moreover, the CSv seems to gather only visual and vestibular information for motor control, but it cannot perform higher-order processing such as sensory integration (Billington and Smith, 2015). Such findings support our results that metabolic changes in the dorsal cingulate cortex and SMA reflect the changes in sensory processing following the onset of postlingual deafness. The sensory-motor interconnection involved in the CSv is attributed to the fact that the dorsal cingulate is directly related to visual processing and undergoes immediate changes following auditory deprivation. On the other hand, the changes in the SMA indirectly connected to the cingulate cortex occur gradually.

### The Dorsoventral Dichotomy Pattern of Metabolism and CI Speech Recovery

We found that glucose metabolism in various areas of the lateral frontal cortex was positively correlated with post-CI speech performance (see Table 4). When a lower statistical threshold (Figure 5B, *p* = 0.01, uncorrected) was applied, areas attaining positive and negative correlations with CI speech perception included the broader regions of the dorsal frontal and ventral cortices. The resting-state activation in the dorsal frontal area increased as the speech perception score increased, whereas the activation in the ventral occipitotemporal region was negatively related to the post CI outcome. However, the dorsal vs. ventral dichotomy of brain metabolism coupled with positive and negative correlation with CI performance was independent of the duration of deafness. This finding replicates the results from our previous resting PET study and fMRI study in congenitally deafened children and postlingually deafened adults (Giraud and Lee, 2007; Lee et al., 2007a; Lazard et al., 2010).

The lateral frontal cortex is associated with higher cognitive functions such as goal-oriented action, top-down modulation (Corbetta and Shulman, 2002), executive control (Koechlin and Jubault, 2006), and working memory (Petrides, 2000). Particularly, this area is known to relate closely with how CI recipients learn electrical signals through the CI. For postlingually deafened CI users who experience a period of auditory deprivation, the learning process is essential to adapt to the artificially processed sounds, which is different from previously acquired auditory sound. To achieve better speech comprehension, CI users need to train themselves to match the spatially degraded CI sounds with intrinsic phonetic representations (Pisoni, 2000; Fu et al., 2005; Moore and Shannon, 2009). In a clinical setting, aural rehabilitation for the matching process is imperative for a successful CI outcome, and a rehabilitation program including training for higher cognitive skills such as good working memory is beneficial for learning CI sounds (Fu and Galvin, 2007; Castiglione et al., 2016). In turn, it is possible that CI users with higher activation in the dorsal frontal regions during the resting state can easily recruit those regions while performing tasks requiring higher cognitive loads. Similarly, previous H_2_^15^O-PET studies have reported that in those with the best CI performance, the prefrontal cortex regions were activated while listening to speech (Giraud et al., 2001; Mortensen et al., 2006; Song et al., 2015). In contrast, CI patients who showed greater brain activation in the ventral temporo-occipital regions, which subserve pattern recognition and long-term memory, may not fully benefit from CI use.

## Conclusion

In summary, we illustrated brain plasticity in adult-onset deafness both in modality-specific and modality-independent areas. The auditory cortex of postlingually deafened patients was more resistant to cross-modal plasticity than that of congenitally deafened people whose decreased metabolic activity in the auditory cortex was significantly related to speech perception performance (Lee et al., 2007a). However, few CI subjects in this study had longer deafness duration than 10 years, which may affect significantly to the correlation results. Thus, clinician who attempt to apply ^18^F-FDG-PET as a biomarker for the CI benefit should use it with a caution.

Until recently, a huge effort has been made to elucidate the clinical relevance of brain plasticity in deafened individuals by examining functional changes in the auditory cortex induced by auditory stimulation with amplification or promontory electrical stimulation (Patel et al., 2007). In this study, we demonstrated that changes occurring in brain areas outside of the primary auditory system appear to be related to speech recovery following CI surgery. Therefore, future studies including tasks regarding visual processing or associated higher cognitive functions would provide useful information to design an optimal rehabilitation program for postlingually deafened CI patients.

## Author Contributions

H-JL, HK, S-HO, and DL designed the research. H-JL and HK performed the research and analyzed the data. H-JL, S-HO, and DL contributed unpublished reagents and analytic tools. J-HH and H-JL wrote the article.

## Conflict of Interest Statement

The authors declare that the research was conducted in the absence of any commercial or financial relationships that could be construed as a potential conflict of interest.
